# Acute symptomatic vitreous floaters assessed with ultra-wide field scanning laser ophthalmoscopy and spectral domain optical coherence tomography

**DOI:** 10.1038/s41598-021-88371-9

**Published:** 2021-04-26

**Authors:** Gisung Son, Joonhong Sohn, Mingui Kong

**Affiliations:** 1 Department of Retinal Service, Hangil Eye Hospital, Incheon, South Korea; 2 Department of Ophthalmology, Catholic Kwandong University College of Medicine, Incheon, 22711 South Korea

**Keywords:** Medical research, Biomarkers, Diagnostic markers

## Abstract

To describe the eyes with vitreous floaters and to analyze the development of acute symptomatic posterior vitreous detachment (PVD). A retrospective review of medical records was performed on patients with the vitreous floater developed for the first time of their life. Peripapillary vitreous opacity (pVO) was searched in Ultra-wide field (UWF) scanning laser ophthalmoscopy and PVD stage was assessed through spectral-domain optical coherence tomography (SD-OCT). 196 patients (55 males and 141 females), who were 58.4 (± 9.1) years old, visited a retinal clinic 9.4 (± 9.1) days after they experienced vitreous floaters. In 196 eyes, pVO was noticed in 122 eyes (62.2%) at UWF. In 106 eyes where SD-OCT data were available, PVD was noticed in 100 eyes (94.3%). Symptomatic eyes showed more advanced stage of PVD (p < 0.001) than symptom free eyes. Eyes with floaters were more myopic (− 0.7 ± 2.2D vs − 0.5 ± 1.9D, p = 0.02), and had lower intraocular pressure (IOP) (14.7 ± 3.2 mmHg vs 15.2 ± 3.0 mmHg, p = 0.02) than the other symptom free eyes. In patients with first floater symptoms, PVD was in progress in most of the eyes not only the symptomatic eyes but also on the contralateral symptom free eyes. Eyes with vitreous floaters were more myopic and had lower IOP than the opposite symptom free eyes.

## Introduction

Vitreous floaters are entoptic images of opacity in vitreous cavity^1^. Substantial portion of patients visit retina clinics complaining symptomatic degenerative floaters. Although floater symptoms often become asymptomatic within a few months from onset, they do cause visual discomfort such as blurred vision, glare and haze in daily living for months or years^2^. Moreover, degenerative vitreous floaters related with posterior vitreous detachment (PVD) could be more clinically significant because they can be followed by pathologic conditions such as vitreous hemorrhage, retinal tear or retinal detachment^3^. Therefore, characterizing the status of PVD could provide important information on eyes with acute vitreous floaters.

Recently, detailed evaluation of vitreoretinal interface has been possible since the introduction of spectral domain optical coherence tomography (SD-OCT)^4–8^. And ultra-wide field retinal imaging (UWF) devices allow us to evaluate posterior pole and wide range of the peripheral retina simultaneously^9^. We hypothesized that the SD-OCT and UWF examination will allow us to identify and record PVD status more accurately. Thus, we investigated patients with acute symptomatic vitreous floaters using these two modalities to determine the relationship between floaters and PVD. By comparing eyes with and without floater symptoms, we also intended to find out when floater symptoms occur on the process of PVD.

## Results

The mean age was 58.38 (± 9.14) years old in average, consisting of 141 women and 55 men (Table 1). The BCVA of 196 eyes was 0.06 (± 0.10) in average, SE was − 0.70 (± 2.09) diopter, and intraocular pressure (IOP) was 15.05 (± 6.20) mmHg. There was no difference between men and women in BCVA (p = 0.64), SE (p = 0.45) and IOP (p = 0.11). In 33 patients (17.3%), vitreous floaters were found in both eyes. Patients visited our retina clinic after an average of 9.41 (± 9.05) days after symptom onset, and 23 patients (11.74%) had ‘flashing light’ symptom before the floater. 24 patients (12.24%) needed laser treatment on peripheral retinal break which was observed in their clinical visit. In 3 eyes (1.53%), glaucoma was diagnosed incidentally and those 3 eyes were excluded from the pRNFL analysis.

**Table 1:** Demographics and ophthalmological outcomes of eyes with acute subjective vitreous floaters occurred within 1 month.

Total of 196 eyes of 196 patients			
Age (21–76)	58.4 ± 9.1 years	Symptom duration	9.4 (± 9.1) days
Sex (M/F)	55/141	Lens status	196 phakic eyes (100%)
Floaters on both eyes	33 pts (17.3%)	Peripheral retinal break	24 (12.2%)
Floaters on unilateral eye	163 pts (82.7%)	Concurrent glaucoma	3 (1.53%)
BCVA (logMAR)	0.06 ± 0.10	**Refractive error**	
S.E. (D)	− 0.7 ± 2.1	Myopic (SE ≤ − 1 D)	61 (31.1%)
IOP (mmHg)	15.1 ± 6.2	Emmetropia (− 1 D < SE < 1 D)	97 (49.5%)
CMT (μm)	260.2 ± 20.7	Hyperopic (1 D ≤ SE)	38 (19.4%)
pRNFL (μm)	101.9 ± 11.6		
**pVO on UWF**		PVD on SD-OCT (*)	106 (eyes total)
None	74 (37.8%)	Stage 0	6 (5.7%)
		Stage 1	8 (7.5%)
pVO	122 (62.2%)	Stage 2	6 (5.7%)
		Stage 3	5 (4.7%)
		Stage 4	81 (76.4%)

Demographics and ophthalmological outcomes of eyes with the first occurrence of acute subjective vitreous floaters that developed within a month. *SD-OCT data for 106 eyes (54%) of the total cohort were available.

*M* male, *F* female, *pts* patients, *BCVA* best-corrected visual acuity, *logMAR* the logarithm of the minimal angle of resolution, *SE* spherical equivalents, *D* diopter, *IOP* intraocular pressure, *CMT* central macular thickness, *pRNFL* peripapillary retinal nerve fiber layer, *pVO* peripapillary vitreous opacity, *UWF* ultra-widefield scanning laser ophthalmoscopy, *PVD* posterior vitreous detachment, *SD-OCT* spectral-domain optical coherence tomography.

**Figure 1:**
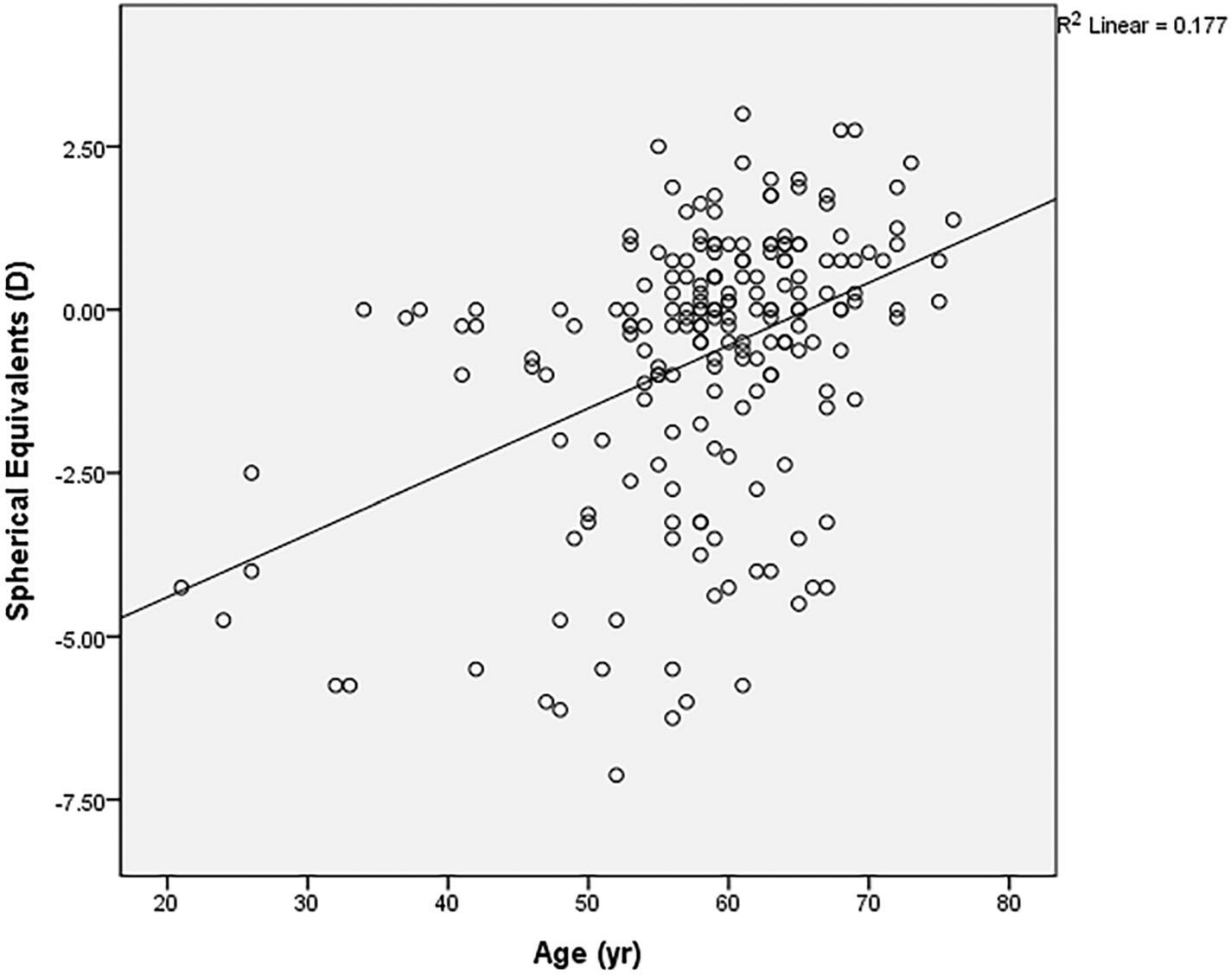
X-axis presents the age when the first vitreous floater symptom occurred, while Y-axis presents the spherical equivalent value in diopters (D). The oblique line of best fit is showing a positive correlation between the spherical equivalent value and age. (r = 0.421, p < 0.001 in the Pearson’s correlation analysis).

Of the 196 eyes with floater, 97 eyes (49.5%) were emmetropic (− 1D < SE < + 1D), 61 eyes (31.1%) were myopic (SE ≤ − 1D) and 38 eyes (19.4%) were hyperopic (SE ≥ + 1D). Myopic eyes tend to present vitreous floater symptoms earlier (r = 0.406, p < 0.001, Fig. 1). This was the same for both the male group (r = 0.352, p = 0.008) and the female group (r = 0.463, p < 0.001). In addition, thinner pRNFL was observed in Myopic patients (r = 0.299, p = 0.011), and SE was not significantly related to central macular thickness (CMT) (r = 0.072, p = 0.471).

Peripapillary vitreous opacity (pVO) was observed in 62.2% (122 out of 196 eyes) in the UWF image. Among 106 symptomatic eyes where SD-OCT data were available, PVD was identified in 94.3% on SD-OCT. PVD was staged based on the morphology of the vitreomacular interphase assessed with SD-OCT, from stage 0 to 4. In 106 symptomatic eyes, 6 eyes were categorized as stage 0, while 8 eyes as stage 1, 6 eyes as stage 2, 5 eyes as stage 3 and 81 eyes as stage 4. Interestingly, almost every eye with the pVO had the evidence of PVD on SD-OCT (98.7%, 75 out of 76 eyes). While, 75.0% (75 eyes out of 100 eyes) of eyes with SD-OCT proven PVD had pVO on UWF images.

We compared the ophthalmologic examination results of the symptomatic study eyes with floaters and the symptom free contralateral eyes without floaters in 163 patients who had symptoms only in the unilateral eye (Table 2). The eyes with floaters were more myopic than those without floaters (− 0.7 ± 2.2D vs − 0.5 ± 1.9D, p = 0.02), and had lower intraocular pressure (14.7 ± 3.2 mmHg vs 15.2 ± 3.0 mmHg, p = 0.02). Visual acuity (0.06 ± 0.11 vs 0.04 ± 0.08, p = 0.09), CMT (259.4 ± 20.2 vs 260.0 ± 19.5, p = 0.56) or pRNFL (100.7 ± 11.6 μm vs 100.5 ± 11.4 μm, p = 0.08) did not differ between the two groups. More pVO on UWF (63.2% vs 18.4%, p = 0.04) and more advantaged PVD stage on SD-OCT (p < 0.001) was observed in the eyes with floater symptoms than the symptom free collateral eyes.

**Table 2:** Comparison between ophthalmological outcomes of eyes with and without vitreous floaters among patients with unilateral symptomatic eyes.

Unilateral vitreous floaters	Symptomatic eyes with vitreous floater (n = 163)	Contralateral eyes without vitreous floater (n = 163)	P-value^+^
BCVA (logMAR)	0.06 ± 0.11	0.04 ± 0.08	0.09
S.E. (D)	− 0.7 ± 2.2	− 0.5 ± 1.9	0.02
IOP (mmHg)	14.7 ± 3.2	15.2 ± 3.0	0.02
CMT (μm)	259.4 ± 20.2	260.0 ± 19.5	0.56
pRNFL (μm) thickness	100.7 ± 11.6	100.5 ± 11.4	0.08
**pVO on UWF**			0.04
None	60 eyes (36.8%)	133 eyes (81.6%)	
pVO	103 eyes (63.2%)	30 eyes (18.4%)	
PVD on SD-OCT (*)	**(86 eyes total)**	**(86 eyes total)**	< 0.001
Stage 0	4 (4.7%)	9 (10.5%)	
Stage 1	6 (6.9%)	12 (13.9%)	
Stage 2	5 (5.8%)	30 (34.9%)	
Stage 3	5 (5.8%)	10 (11.6%)	
Stage 4	66 (76.7%)	25 (29.1%)	

*PVD was staged according to SD-OCT finding as Stage A (perifoveal), Stage B (macular PVD with vitreopapillary adhesion), and Stage C (complete PVD). The SD-OCT data for 53% (86 out of 163 eyes) were available.

^+^P-value was calculated using the Student *t* test for numerical values and Fisher exact test for categorical values.

*BCVA* best-corrected visual acuity, *logMAR* the logarithm of the minimal angle of resolution, *S.E.* spherical equivalents, *D* diopters, *IOP* intraocular pressure, *CMT* central macular thickness, *pRNFL* peripapillary retinal nerve fiber layer, *pVO* peripapillary vitreous opacity, *UWF* ultra-widefield scanning laser ophthalmoscopy, *PVD* posterior vitreous detachment, *SD-OCT* spectral domain-optical coherence tomography.

We could verify a high degree of agreement on the interpretation of UWF and SD-OCT images between two investigators (GSS, MGK) In UWF image analyses, the judgments were in agreement in 190 eyes (96.9%). Regarding SD-OCT image analyses, the ICC value between the two investigators was 0.977. There were six discordant interpretations on UWF images of myopic eyes with tessellated fundus. And there were three disagreements on SD-OCT images where background reflectivity of vitreous cavity was difficult to be determined as vitreous body or not.

Additional ophthalmological analyses were performed on 33 patients with floater symptoms on both eyes (Supplementary Table 1). The comparison was conducted between eyes with earlier floater symptoms versus eyes with more recently occurred floater symptoms. BCVA, SE, IOP, CMT and pRNFL thickness were not different each other (p = 0.48, 0.39, 0.24, 0.65, 0.71 respectively). The prevalence of the pVO on the UWF were not different between 2 groups, (57.6% vs 36.4, p = 0.505) neither so PVD stage (p = 0.90).

## Discussion

This study provides novel information regarding the vitreoretinal interface of patients experiencing their lifetime’s first floater symptom. Meanwhile, through this study, we were able to confirm several facts that have been reported about PVD in previous studies. First, the first vitreous floater in life showed at an average age of 58.4 years, similar to the previous studies^10–13^. The female predominance of PVD in this study (72% in female) has been also reported several times in previous studies^10,13^. In addition, the earlier onset of PVD in the myopic eye in this study has also been reported in several other previous studies^10,14,15^.

The usefulness of SD-OCT in the diagnosis of PVD was recently presented in a previous prospective study. Moon et al.^16^ analyzed 124 eyes with PVD examined using ultrasonography and SD-OCT and showed the usefulness of adding peripapillary OCT scans in the diagnosis of PVD considering lower inter-examiner agreement of ocular ultrasound scanning. Our data could be convincing evidences supporting their hypothesis. Instead of ultrasonography, however, we described the inter-correlation of UWF and SD-OCT assessing eyes with vitreous floaters. We believe that our study could describe more ‘real world’ clinical situation than their data analyzed from prospectively enrolled patients.

Most of the eyes with floaters had PVD in progress, and the proportion was 94.3% as determined by the SD-OCT. Interestingly, PVD was also progressing in most of the asymptomatic eyes. We confirmed the evidence of PVD assessed with SD-OCT in 89.5% of the symptom free contralateral eyes. This inconsistency between presence of PVD on SD-OCT and absence of subjective symptoms could be explained by the PVD stage of the opposite symptom free eye which was earlier than the other symptomatic eye. In other words, the degree of detached membrane might be relatively small to be noticed in earlier PVD stage. Since this study is cross sectional, it was difficult to accurately determine at which point the floater occurred during the PVD process. However, we could confirm that the eyes with floater present more advanced PVD stage on SD-OCT and more eyes with pVO on UWF than the symptom free contralateral eyes.

The fact that the eyes with floaters were more myopic and had lower IOP can be explained in relation to changes in the eyeball as PVD progresses. Myopic eyes have longer axial length, so maybe easily affected by the shrinkage of the vitreous body. We can also infer that a more myopic eye in a single person might expect earlier vitreous floaters than the other eye. Meanwhile, the measurement of low intraocular pressure in PVD eyes can be explained by the fact that stiffer vitreous in eyes without PVD may present a stronger rebound than liquefied vitreous in eyes with PVD during the measurement of IOP.

Peripapillary vitreous opacity presented on UWF could be thought as a convincing evidence of PVD. This can be intuitively understood, because the pVO is likely to be found in UWF after PVD initiation. Weiss ring, which is the representative form of pVO in the advanced stage PVD, is noticed when complete PVD occurs after the detachment of the vitreopapillary adhesion. Also in this study, almost every eyes with visible pVO were proven to have PVD through SD-OCT examination. For a few exceptional cases (2 eyes, 2.67%), pVO could be positioned perpendicular to the front, difficult to be photographed. Considering this, if Weiss ring is observed in UWF, ophthalmologists who are practicing in the environment without SD-OCT equipment could be more confident in the presence of the PVD.

Based on this study results, retinal examinations of both eyes are recommendable on the patients complaining of floaters. This is because described above, PVD was occurring in 89.5% of the contralateral eyes even without subjective floater symptoms. If early-staged PVD findings on SD-OCT are observed in the opposite eye, the patient can be explained in advance that floater symptoms may also occur in the symptom free eyes. In addition, in 6 eyes (1.95%) of the study cohort, a peripheral retinal break requiring laser photocoagulation treatment was found in the opposite eye rather than in the symptomatic eye.

Despite high degree of agreement, discrepancy in the interpretation of UWF and SD-OCT tests between the two investigators should not be ignored. In 6 UWF images, there was a disagreement whether the spots on the photos to be judged by vitreous opacity or peripapillary pigments. The ambiguity got bigger in myopic eyes with tessellated fundus. In 3 SD-OCT images, it was difficult to determine whether PVD stage be categorized into stage 1 or stage 2. Serial checks-up of the test, correlations with the stereoscopic fundus examination findings or performing swept-source OCT covering more extensive retina could be helpful approaches for solving the discrepancy.

Although it is useful to examine UWF and SD-OCT together in patients with vitreous floaters, yet detailed medical history and meticulous retinal observation through a microscope should not be overlooked. This is because floater symptoms are not necessarily caused by PVD. If we listen to the patients carefully through detailed medical history, we can better understand the patients-reporting ‘floaters’ symptoms. Thorough retinal examination should be performed to conclude whether vitreous opacities are related with PVD or not.

We are mindful of the limitations of this study, such as its retrospective and cross-sectional nature performed on single-ethnic population background. In addition, section bias should be mentioned as we analyzed only the patients who visited clinic. In addition, SD-OCT was performed on 106 patients (53%) who were willing to pay for the examination. However, as we know of, this is the first attempt to analyze lifetime’s first vitreous floaters using both of the UWF scanning laser ophthalmoscopy and the SD-OCT images. We believe our study might provide useful information to ophthalmologists regarding vitreous floaters and PVD in real clinical world. Further prospective studies might warrant for more useful information of when and in what percent the vitreous floater symptoms fades away.

## Methods

**Figure 2:**
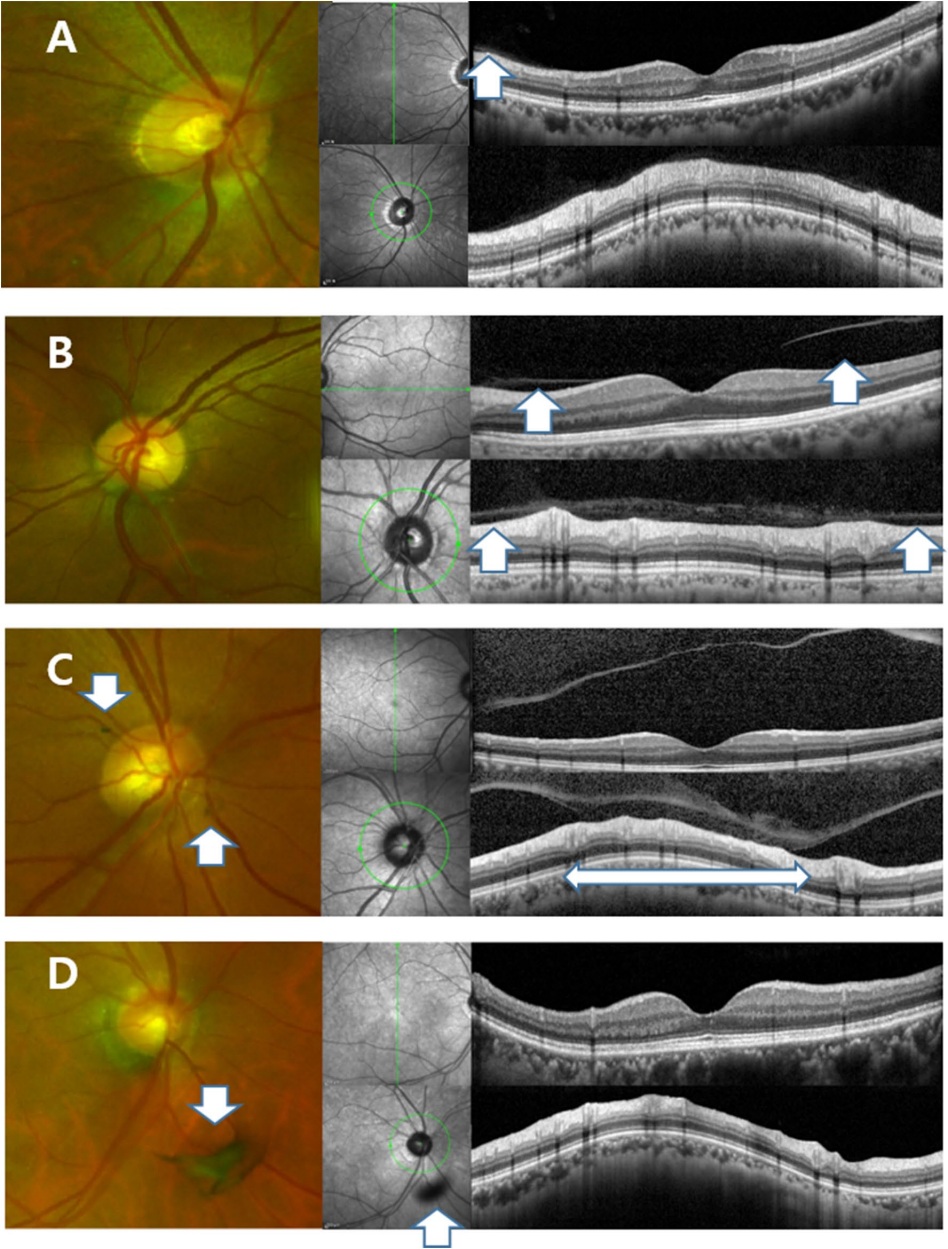
Ultra-wide field scanning laser ophthalmoscopy (UWF) and Spectral Domain Optical Coherence Tomography (SD-OCT) images of patients who experienced floater for the first time in their life. (**A**) PVD stage 1. 59 year-old male. No definite change was noticed around optic disc in UWF. Note subtle change which is presenting the initiation of PVD (Posterior Vitreous Detachment, white arrow). (**B**) PVD stage 2. 60 year-old female. No definite change was noticed around optic disc in UWF. In the macular SD-OCT image, perifoveal posterior vitreous detachment (PVD) is in progress (white arrows) remaining posterior vitreous attached to fovea. In glaucomatous SD-OCT, PVD is in progress in some part of the optic disc margin (arrow), while most of the optic disc remains attached to the vitreous body. (**C**) PVD stage 3. 61 year-old male. Note two pVOs (peripapillary vitreous opacity, white arrows) noticed around the optic disc. In the macular SD-OCT image, PVD occurred throughout the entire macula. In the glaucomatous SD-OCT image, PVD is in progress while about half of the optic disc margin is detached to the vitreous body (a wide bidirectional arrow). (**D**) PVD stage 4. 66 year-old female. Note a dense pVO (white arrow) resembling a Weiss ring. In the macular SD-OCT image, reflectivity of vitreous body is not observed in hollow vitreous cavity. In glaucomatous SD-OCT image, reflectivity of vitreous body is not observed either. A shadow of pVO (arrow) is identified.

We retrospectively analyzed the medical records of patients who visited our retina clinic due to newly developed vitreous floaters within a month from July 2017 to May 2020. All patients received thorough ophthalmological examination including BCVA, manifest refraction, IOP (NT-530P, Nidek, Aichi, Japan), retinal examination after mydriasis, SD-OCT (Version 5.3.2.0; Heidelberg Engineering, Heidelberg, Germany) and UWF (Optomap, Optos PLC, Dunfermline, Fife, Scotland, UK) were performed. We conducted SD-OCT line and raster scan in all eyes centered at the fovea. Automatic real-time (ART) mode was activated when horizontal and vertical line scans were conducted with 25 frames averaged. In the raster scan image, a 30 × 25 degree^2^ area was covered with 31 b-scans (consisting of 768 A-scans), which are 9.0 mm in length, and spaced at 240 μm apart. The peripapillary region was scanned using a circular scan 12° in diameter, centered on the optic disc (Fig. 2). Every retinal examination was performed on both eyes. All the images information was assessed by saved data on digital files before in-depth analyses.

### Study subjects and vitreous floaters

Eligible subjects were adults older than 20 years old who experienced acute symptom (onset ≤ 1 month) of vitreous floaters for the first time of their life. In patients with floater symptoms in both eyes, eyes with more recently developed floater symptom were selected for the analysis. Exclusion criteria includes history of ocular surgery including cataract operation, history of ocular injections, any retinopathy including diabetic retinopathy or retinal vein occlusion, medial opacity including severe cataract or corneal opacity or eyes with obscure image which were not suitable for proper analyses. Eyes received refractive surgeries were excluded from the refraction analyses.

In this study, vitreous floaters were defined as symptoms of unprecedented amorphous ‘floating’ material. Other confusing symptoms such as fixed spots interrupts visual axis, transient wavy visual disturbance or metamorphopsia were excluded with through history taking. Concurrent subjective symptoms such as headaches, flashes, or ocular pain were asked also.

### Analyses of ophthalmologic outcomes

Best-corrected visual acuity was measured with the manifest refraction test and was recorded in logMAR (logarithm of minimal angle of resolution). The spherical equivalent and cylindrical value were expressed in diopter. CMT at the 1-mm center of the fovea was measured using a built-in software (Heidelberg Eye Explorer, version 1.10.2.0, Heidelberg Engineering, Heidelberg, Germany), which recorded the distance between the vitreoretinal surface and the border between the retinal pigment epithelium and the Bruch’s membrane. Peripapillary RNFL (pRNFL) thickness was measured using the identical software.

### Analysis of peripapillary vitreous opacity in UWF imaging

The presence of pVO was observed in UWF images taken on the eyes, after pupil dilation. The vitreous opacity found within 3 disc diameters from the center of the disc was included (Fig. 2). In cases where it was difficult to judge by pictures alone, the precise fundus examination with indirect ophthalmoscopy was considered together for the determination. The two investigators (GSS, MK) each independently analyzed the image, and for the inconsistencies, the senior investigator (JHS) made a final decision.

### Anaylsis of PVD stage in SD-OCT imaging

The staging of PVD was performed base on the previously published paper^17^. In brief, stage 0 = no PVD; stage 1 = PVD at mid-periphery and possible subtle PVD in the posterior retina; stage 2 = PVD, except for persistent adhesion to the papilla and fovea; stage 3 = PVD, except for persistent adhesion to the papilla; and Stage 4 = complete PVD (Fig. 2). The two investigators (GSS, MK) analyzed PVD respectively, and the opinions of senior investigator (JHS) were sought for discordant findings.

### Statistical analyses

Continuous values were expressed as “average ± standard deviation,” and categorical variables were described as proportions; differences between groups were determined using the Chi-squared test or Fisher’s exact test. Comparison of ophthalmological values between eyes with/without vitreous floaters was performed using the paired *t* test. Pearson’s correlation analyses were performed among various continuous values. Inter-class correlation (ICC) value was calculated to determine how consistent the two investigators’ interpretations on PVD at SD-OCT images were. All data were inserted into an Excel spreadsheet (Microsoft Corp.) and analyzed using SPSS software (version 23; IBM Corp, New York, NY). A P-value less than 0.05 was considered statistically significant.

### Ethics approval and patient consent

This study was approved by the Institutional Review Board (IRB) of Hangil Eye Hospital (IRB number: IRB-20006) and complied with the Declaration of Helsinki in conducting the study. Given the retrospective design of this study and the use of anonymized data, requirements for informed consent were waived by the IRB.

## Supplementary Information

Supplementary Table 1.
