# Supplementary material for: Acute symptomatic vitreous floaters assessed with ultra-wide field scanning laser ophthalmoscopy and spectral domain optical coherence tomography
Source: Sci Rep. 2021 Apr 26;11:8930. doi: 10.1038/s41598-021-88371-9 (PMC8076170; doi:10.1038/s41598-021-88371-9)
Supplement: Supplementary file 1 — Supplementary Table 1. [file 41598_2021_88371_MOESM1_ESM.pdf]

Acute Symptomatic Vitreous Floaters Assessed with Ultra-Wide Field Scanning Laser Ophthalmoscopy and Spectral Domain Optical Coherence Tomography

Gisung Son, M.D.[1,2], Joon Hong Sohn, M.D.[1], Mingui Kong, M.D.(*) [1,2]

1. Department of retinal service, Hangil Eye Hospital, Incheon, South Korea

2. Department of Ophthalmology, Catholic Kwandong University College of Medicine, Incheon 22711, Korea

Corresponding author

Mingui Kong, M.D.

Department of Ophthalmology, Hangil Eye Hospital

35 Bupyeong-daero, Bupyeong-gu, Incheon, Korea. 21388

Tel: +82-32-503-3322

Fax: +82-32-504-3322

E-mail: eyedockong@gmail.com

Supplementary Table 1.

Comparison between the ophthalmological outcomes of the two eyes of patients who complained of floaters in both eyes

Patients with unilateral vitreous floaters	Earlier Sx eyes * (n = 33)	Later Sx eyes * (n = 33)	P-value⁺
Age (21~72)			N/A
BCVA (logMAR)	0.04 ± 0.06	0.06 ± 0.13	0.48
S.E. (D)	-00.66 ± 1.76	-1.26 ± 3.66	0.39
IOP (mmHg)	17.03 ± 13.34	14.21 ± 3.18	0.24
CMT (㎛)	262.44 ± 24.35	258.94 ± 21.24	0.65
pRNFL thickness (㎛)	104.60 ± 11.73	105.60 ± 0.51	0.71
Weiss ring on UWF			
- None	14 eyes (42.4%)	21 eyes (63.6%)	0.51
- Weiss ring	19 eyes (57.6%)	12 eyes (36.4%)	
PVD on SD-OCT (*)	(20 eyes in total)	(20 eyes in total)	
- None	2 eyes (10.0%)	3 eyes (15.0%)	
- Stage A	3 eyes (15.0%)	3 eyes (15.0%)	0.90
- Stage B	Absent	Absent	
- Stage C	15 eyes (75.0%)	14 eyes (70.0%)	

Comparisons between both eyes of patients with bilateral vitreous floaters.

* Eyes were divided into earlier and later classes depending on which eye developed the vitreous floaters first.

⁺ P-value was calculated using students' T-test for numerical values and Fisher's exact test for categorical values.

Sx: symptoms; BCVA: Best-Corrected Visual Acuity; logMAR: logarithm of minimal angle of resolution; S.E: Spherical Equivalents; D: Diopters; IOP: Intraocular Pressure; CMT: Central Macular Thickness; pRNFL: peripapillary Retinal Nerve Fiber Layer;
